# Editorial: Insights in extreme microbiology: 2021

**DOI:** 10.3389/fmicb.2022.1119051

**Published:** 2023-01-04

**Authors:** Andreas P. Teske, Virginia P. Edgcomb

**Affiliations:** ^1^Department of Marine Sciences, University of North Carolina at Chapel Hill, Chapel Hill, NC, United States; ^2^Department of Geology and Geophysics, Woods Hole Oceanographic Institution, Woods Hole, MA, United States

**Keywords:** insights 2021, Research Topic, editorial, extreme microbiology, diversity

This Research Topic has turned into an experiment. Without prescribing a specific research area, and in doing so, giving carte blanche to the associate and review editors of the Extreme Microbiology section, we wondered who would respond, and what kinds of papers would be submitted? Now it is time to wrap up the experiment and to take stock. Research teams from eight countries (USA, Japan, China, India, South Korea, Germany, France, Argentina) published fifteen articles, eight of these by female first authors and/or senior authors. Three author teams have taken the opportunity to write synthesis papers and to formulate current and future perspectives on hydrothermal vent and subsurface microbiology. While the contributions to this special topic reflect the wide-ranging endeavors and the extensive scientific expertise of our associate and review editors, some shared themes are emerging. Hydrothermal vent microbiology emerges as one of the best-represented fields and accounts for seven articles; recognizably related themes are taken up by a review on the deep terrestrial subsurface. Five physiological studies explore individual protein function or complex gene expression responses in obligate or facultative extremophiles, and two studies explore spatially or temporally changing marine microbial assemblages. Without further ado, here is the harvest of the year 2021.

In their hypothesis and theory article, Edgcomb et al. discuss the potential roles of hydrocarbon-degrading fungi and sulfate-reducing bacteria, and the possibility that they may interact in the hydrocarbon-rich hydrothermal sediments of Guaymas Basin to degrade specific hydrocarbons. While numerous lineages of alkane- and aromatics-degrading bacteria and fungi coexist in this habitat, the study of their interactions, for example in sequential or syntrophic hydrocarbon degradation, is just beginning.

The massive sediments of Guaymas Basin were recently probed by deep drilling during IODP Expedition 385. In surveying the activity of sulfate-reducing bacteria in diverse locations across Guaymas Basin, Nagakura et al. find activity peaks linked to hydrothermal influence and steep thermal gradients at the off-axis hydrothermal Ringvent site, where sulfate-reducing activity exceeds even levels at cold seep locations.

The microbial communities of hydrothermal vent sites are increasingly targeted with a complex network of *in-situ* and *ex-situ* analyses, stable isotope probing, geochemical and microbial process studies, (meta)genomic sequencing, and functional annotation of uncultured microbial lineages, and biogeochemical rate measurements and modeling. As discussed by Böhnke and Perner, these multipronged strategies are aiming for a holistic understanding of hydrothermal vent ecosystem function, a goal that becomes increasingly realistic.

Numerous hydrothermal vent locations remain poorly studied, in particular those in the remote reaches of the Southern Indian Ocean. Metagenomic analyses of hydrothermal chimneys of the Southwest Indian Ridge have uncovered novel types of sulfur-oxidizing bacterial communities where members of the gammaproteobacterial *Ectothiorhodospiraceae* predominate. Based on the discovery of bacteriophytochrome-encoding genes in some members of the Thiohalomonadales from this location, Wang et al. speculate on their potential for absorbing infrared radiation.

Hydrothermal locations do not have to be marine. In their sequence-based study of benthic mat communities at hydrothermal springs in Crater Lake (Oregon), Stromecki et al. find chemosynthetic bacteria that thrive by aerobic oxidation of iron, sulfur and nitrite; anaerobic pathways including arsenic reduction and denitrification; carbon fixation relies on the reverse TCA and the reverse pentose phosphate cycles. The importance of iron oxidation, and the prevalence of iron-oxidizing bacteria (*Gallionella*, Zetaproteobacteria) links these freshwater hydrothermal communities to iron-rich hydrothermal communities at Loihi seamount, and separates them from the vast majority of marine hydrothermal sites.

The cultivation and physiological study of extremophilic bacteria from deep-sea hydrothermal vents has always been hampered by the impossibility or at least difficulty of recreating hydrothermal vent conditions in the laboratory. Cario, Larzillière et al. introduce a miniaturized high-pressure microfluidic device that allows testing the temperature and pressure responses of deep-sea hyperthermophiles without depressurization, and they demonstrate this methodology with the barophilic model strain *Thermococcus barophilus* and the hydrogenotroph *Methanothermococcus thermolithotrophicus*. In a related study Cario, Oliver et al. compare survival and growth of a mesophilic deep-subsurface sulfate-reducing bacterium (*Desulfovibrio*) and a hyperthermophilic deep-sea archaeum (*Archaeoglobus*) under isobaric high-pressure cultivation, and under cyclic high-pressure cultivation followed by decompression. They note that sample decompression impacts growth negatively for both test organisms; since this effect becomes more severe under non-optimized pressure conditions. Depressurization selects against the enrichment and isolation of piezophiles. This difficulty can be circumvented using isobaric enrichment and culture methods, such as microfluidic devices with multiple cultivation chambers lined up along adjustable thermal and chemical gradients.

Terrestrial subsurface microbiology has developed as a sister field to marine deep subsurface and hydrothermal vent research; both fields share themes of rock-hosted energy and carbon sources that sustain subsurface microbial life independently from the surface biosphere. Meyer-Dombard and Malas survey recent progress in the study of microbial life and microbial ecosystems in deep rock laboratories, mines and boreholes, and introduce numerous case studies where microbial communities are sustained by iron and sulfur in deep basalt crust, or by hydrogen-generating serpentinization reactions. At the confluence of cracks in the rock, water and energy sources, microbial life is never far.

Physiological studies of extremophilic microorganisms constitute the next major group of papers in this special topic, represented by three articles. The hyperthermophilic archaeum *Thermococcus onnurineus* relies on formate dehydrogenase gene fdh2 for formate-dependent growth and mediation of NAD^+^-dependent formate oxidation. By purifying and testing the function of formate dehydrogenase gene fdh3, Yang et al. show that this hydrogenase mediates electron transfer between formate and NADPH or ferredoxin, indicating redox-dependent finetuning of formate dehydrogenase activity.

An alkaliphilic *Microbacterium* strain with unusually high tolerance to the toxic alkali metal cesium (1.2 mM, almost twice the previously known limit of 0.7 mM), isolated from jumping spider ground extract, contained a novel low-affinity Cs^+^/proton antiporter that removed Cs^+^ from the cytoplasm. Koretsune et al. discuss the potential of this cesium-resistant bacterium and its antiporter as a bioremediation tool for purification of water contaminated with cesium radionuclides.

In a bioenergetic study of the obligate alkaliphile *Evansella clarkia*, a member of the Bacillaceae, Goto et al. demonstrate a new strategy to overcome the limitations of high extracellular pH for chemiosmotic energy generation. The predominant membrane-bound cytochrome C contains an extra asparagine-rich segment between the membrane anchor and the main body of the protein which appears to form a H^+^ network that accumulates protons on the outer membrane surface. Thus, *E. clarkii* constructs a proton capacitator that ensures sufficient transmembrane proton flux and energy generation even under low-oxygen, high-pH conditions.

Two studies highlight the complex physiological adaptations of bacteria to high altitude habitats, where microbes have to contend with cold and dry conditions, and high UV exposure. Zannier et al. investigate the impact of extreme UV irradiation on the proteome of a *Nesterenkonia* strain, revealing its oxidative stress response, DNA damage repair, and reallocation strategies for metabolic energy that allow this actinobacterium to survive chronic UV stress in the Andean high plateau; the resulting network of physiological responses has been termed the “UV-resistome”. In a study by Kumar et al., high-altitude stress conditions (desiccation, extreme temperature fluctuations, UV exposure) also impact the lifestyle of a *Iodobacter* strain from a Himalayan lake; the bacterium is responding with custom-tailored survival strategies, including synthesis of antifreeze proteins and protective pigment formation.

Although Eukaryotes do not inhabit the same environmental extremes as bacteria and archaea, they do occur in abundance and in great diversity in some extreme habitats, for example in the polar oceans. Grattepanche et al. provide an example from the Antarctic ocean, a survey of eukaryotic phyto- and zooplankton along the Western Antarctic Peninsula in southern spring. Diatoms dominated the phytoplankton, and phototrophy was generally the dominant trophic mode. Toward the south, mixotrophic, phagotrophic and parasitic communities increased, and in the southernmost station nanoplankton predators dominated. Climate change will likely shift this phototrophic-heterotrophic community gradient further south.

We conclude this Research Topic with a paper by Kevorkian et al. on methane cycle dynamics at the marine methane-sulfate interface. Depending on the availability of electron donors and acceptors, methanogenesis and anaerobic methane oxidation intersect at this interface, where both processes appear to be mediated by ANME-1 archaea. In long-term incubations that transition from sulfate reduction to methanogenesis, ANME-1 archaea are selected against, methane is cycled at low sulfate concentrations for several months, followed by steady growth of sulfate reducers along with methanogens after sulfate is depleted. These results indicate a complex network of changing interactions between sulfate-reducing bacteria and methanogens.

To summarize, “*Insights in extreme microbiology 2021*” showcases not only the diversity of themes and contributors but also the different research scales in Extreme Microbiology, from individual proteins and enzymes to global microbial ecosystems. Of course, fifteen papers are just a start, and some readers may already miss their favorite topics, for example acidophiles, halophiles, or Astrobiology. To broaden the scope and to provide further opportunities, we note that the “*Insights in extreme microbiology 2022*” Research Topic is open for manuscript submission at the time of writing.

## Author contributions

AT and VE wrote and edited the editorial. Both authors contributed to the article and approved the submitted version.

